# Real-world evidence for postgraduate students and professionals in healthcare: protocol for the design of a blended massive open online course

**DOI:** 10.1136/bmjopen-2018-025196

**Published:** 2018-10-04

**Authors:** Edward Meinert, Abrar Alturkistani, Josip Car, Alison Carter, Glenn Wells, David Brindley

**Affiliations:** 1 Department of Paediatrics, University of Oxford, Oxford, UK; 2 Department of Public Health and Primary Care, School of Public Health, Imperial College London, London, UK; 3 Department of Infectious Disease Epidemiology, Faculty of Medicine, School of Public Health, Imperial College London, London, UK; 4 Oxford Academic Health Science Centre, Oxford, UK

**Keywords:** massive open online course, real world evidence, real world data, data science

## Abstract

**Introduction:**

There is an increased need for improving data science skills of healthcare professionals. Massive open online courses (MOOCs) provide the opportunity to train professionals in a sustainable and cost-effective way. We present a protocol for the design and development of a blended MOOC on real-world evidence (RWE) aimed at improving RWE data science skills. The primary objective is to provide the opportunity to understand the fundamentals of RWE data science and to implement methods for analysing RWD. The blended format of MOOC will combine the expertise of healthcare professionals joining the course online with the on-campus students. We expect learners to take skills taught in MOOC and use them to seek new employment or to explore entpreneurship activities in these domains.

**Methods and analysis:**

The proposed MOOC will be developed through a blended format using the Analysis, Design, Development, Implementation and Evaluation instructional design model and following the connectivist–heutagogical learning theories (as a hybrid MOOC). The target learners will include postgraduate students and professionals working in the health-related roles with interest in data science. An evaluation of MOOC will be performed to assess MOOCs success in meeting its intended outcomes and to improve future iterations of the course.

**Ethics and dissemination:**

The education course design protocol was approved by EIT Health (grant 18654) as part of the EIT Health CAMPUS Deferred Call for Innovative Education 2018. Results will be published in a peer-reviewed journal.

**Strengths and limitations of this study**

- The strengths of the blended massive open online course design is the use of pedagogical design based on the connectivist and heutagogical learning theories.
- Another strength is that the evaluation study of the course is embedded in the course design increasing the likelihood of a timely evaluation.
- Successful implementation and evaluation of the course will depend on learner recruitment and retention.

## Introduction

Healthcare is becoming increasingly dependent on data analytics for improvement of care, efficiency and quality, and getting more value from resources.^1^ However, the fast-growing data analysis field requires constant and up-to-date training in new skills.^2^ There is a high demand for trained data scientists in healthcare,^1^ as health is associated with challenges that can be solved by advanced data science methods with the potential to improve delivery of patient outcomes by increasing efficiency and effectiveness. For example, using big data methods on the vast quantity of real-world data (RWD) enables providers the ability to create predictive models which can help accurately identify risk of disease and improved and personalised preventive care for patients.^3^ RWD includes the vast quantity of data that falls outside the boundaries of controlled clinical trials which can be used for: measuring healthcare resource-use and the burden of disease, evaluating prescribing patterns or clinical outcomes of a new medicine, describing current treatment patterns for a patient group for baseline information.^4^ Real-world evidence (RWE), which is derived as a result of RWD, is starting to play a significant role in decision-making, and it is expected to grow in the future.^3^

Mentoring, team-based training and self-study are recognised as effective measures to train professionals in data analytical skills.^2^ Massive open online courses (MOOCs), a form of web-based online learning, provide skills adoption opportunities through the provision of learning in an open, publicly available platform that is often free of charge on specialised topics.^5^ MOOCs can increase the knowledge of professionals in a new topic^6^ and can help them gain new skills.^7^ MOOCs have gained large popularity in different fields in the last 10 years,^8^ but despite the availability of numerous MOOCs on data science, currently, there are very limited courses focusing on the topic of RWE. Also, there has been limited, but increasing use of MOOCs in a blended capacity and use of this project’s proposed instructional design shall create the opportunity to observe how a MOOC can be managed in this capacity, considering the logic and structural issues incumbent in this format.^9^

Dealing with RWD can be daunting and challenging due to its volume, scale and the requirement to understand the source data and its collection in context. Researchers and healthcare professionals who want to use RWD need to learn new methodologies and frameworks which deviate from standard research methods. Although MOOCs provide an opportunity to train healthcare professional in data science skills, very limited number of MOOCs have touched on the topic of RWE. This MOOC will therefore create an educational programme that focuses on RWD data analysis methods and skills to improve healthcare delivery.

The proposed blended-MOOC will prepare future (postgraduate students) and current healthcare professionals with the required skills and knowledge to conduct and be part of RWE projects. Through the course, we aim to impact the learners’ knowledge, skills and attitudes on the use of RWE data science in healthcare, as well as their communication and participation in networks of data science in healthcare. We also aim to inspire the use of RWE methods across various postgraduate curriculum disciplines, National Health Service (NHS) commissioning support organisations, healthcare regulation organisations and life sciences industries (ie, pharmaceuticals, biotechnology and medical devices). RWE MOOC was funded in April 2018. Planning preparation and planning for the course will begin in June 2018 with the identification of key themes and concepts to be translated into content. Course production will commence in June 2018, and the course will go live in December 2018. The course will be deployed on the Innoenergy X-KIC FutureLearn Platform. The impact and efficacy of this delivery model will be evaluated as a mixed-methods study; further details of the evaluation protocol are defined in a separate publication.^10^

We aim to bring the online learning component (MOOC) together with a face-to-face university level course, to enhance the learning experience and to challenge and enrich postgraduate-level course taught with traditional methods. This will allow diversifying knowledge delivery mediums and will enable university students to be exposed to a global community of learners. MOOC will allow learners the flexibility of amending the learning from any location, the advantage of meeting a large number of students outside their regular university classrooms and will meet EIT Health’s call for ‘massive dissemination’ of innovative education ideas. A blended course will allow knowledge integration between the university students and the global learners joining MOOC from all over the world which can add richness and enhance the learning experience for all learners. The limited availability of courses on RWE, a topic that is gaining popularity in healthcare and the use of the latest advances in online education (blended MOOC), is what makes our proposed programme unique regarding the topic and educational strategies used.

## Methods and analysis

### MOOC overview

The project will deliver an education programme via a MOOC and a face-to-face course, to create a blended format for postgraduate students and professionals interested in the application of RWE data analysis and in furthering their knowledge base and skill-set to include conducting and commissioning RWD analysis. To help meet this goal, a key objective of MOOC is to establish a global network of people to continue and advance the dialogue on data science in healthcare. It is expected that MOOC will provide the opportunity to understand the fundamentals of RWE data science and to implement methods for analysing RWD.

### MOOC development

The course will be organised from Imperial College’s Global eHealth Unit, and course material will be filmed on Imperial College premises. The face-to-face component of the course will be offered at the University of Oxford. The course will be delivered in 5 weeks (five modules). However, the fifth module will only be offered to learners who sign up for certification. The course will be offered on specific dates and will be run for 5 weeks, and each module will be released on a weekly basis. Pilot implementation of the course will occur in 2018; partners will construct the core course programme, developing the face-to-face course and the complementary digital learning. Two face-to-face workshops will be held in the UK to pilot the blended course design. Scale-out will occur in 2019, face-to-face workshops will be held in France, Sweden, Spain and the UK, one per quarter. This will expand the reach of the programme and facilitate project development which will be done in conjunction with industry partners in each European Union region. Workshops will include industry partners focused on the design, development and implementation of RWE methods.

### MOOC pedagogical design

Course design will be based on the hybrid connectivist–heutagogical learning theories. The connectivism theory emphasises the use of networking and communication to deliver the learning material, where interactions and discussions between learners can also contribute to the introduction of new knowledge.^11^ Heutagogy principles emphasises creating competent learners through touching on ‘learner autonomy and self-directedness’.^12^ To enhance the connectivist characteristics of course, the digital platform will be created in a way that can enable learners to create social networks and communicate with like-minded individuals. One of the objectives of the course is to establish a global network to continue and advance the dialogue on data science in healthcare. Attendance in a course with healthcare professionals from around the world will give the postgraduate students the opportunity to appreciate the role of data in real-life clinical settings and introduce them to methods and challenges faced in practical healthcare settings. At the same time, healthcare professionals joining the course will be able to communicate and engage with other professionals who will be possibly using data in a different healthcare setting; clinical or non-clinical. The heutagogical characteristics of the course will be enhanced, by allowing learners the flexibility to go through the course material in their own time and to move through the material according to their preference. This learning principle will also be enhanced by incorporating social media into the discussions,^12^ and creating a course hashtag that can be accessed by learners for further discussion.

### Teaching strategies

The course plan will be prepared following the Analysis, Design, Development, Implementation and Evaluation (ADDIE) model (instructional design model).^13^ The ADDIE model provides a systematic, step-by-step framework to ensure the course is developed in a structured and comprehensive way.^14^ To teach innovation, learners will be structured into teams to develop and implement a data science project. These initiatives will be RWE projects that will address current health problems. Our assessment strategies will include both formative (polls and peer-assessment) and summative assessments (multiple choice questions/module). We will offer an optional fee-based certificate for completion of MOOC and a separate project. The project will involve the proposal of an RWE data analysis methodology (using techniques learnt in the course) to solving a current healthcare problem. Learners will be engaged in collaborative exercises and reflective discussions with other learners where the ‘teacher’ will act as a facilitator and a contributor to the discussions. Each module will provide a list of required reading, recommended reading and RWE-related datasets and tools to allow learners to individualise their learning and explore other sources of knowledge.^15^

### Target learners

The course will cater to two types of learners: current university-level students (postgraduate) and professionals in the healthcare field (eg, professionals working in healthcare, commissioners, policy-makers, healthcare regulators and those working in the life sciences) (1) who have some background knowledge of statistics in healthcare and/or (2) who carry out hands-on data analysis in their work or studies. The target audience will include postgraduate students in health-related fields with an interest in the use of RWD and RWE data science in healthcare and professionals working in NHS commissioning, healthcare regulation and life sciences. Selection parameters will be based on participant’s occupation and statistical knowledge. Participants who are postgraduate students or professionals in healthcare fields, who have completed a basic university-level statistics course or who have professional experience in data analysis and research will be eligible to join MOOC. The required prerequisite is knowledge in basic statistics and understanding of statistical measures used in healthcare which is met if the learner completed a university-level basic statistics course or professionally works on data analysis.

### Learning outcomes

The content from the course develops skills in the context of RWE, including frameworks for analysing and evaluating content (data) and subsequently, will encourage them to embark on healthcare RWE-related projects, innovation and entrepreneurship. To complete the course, learners will be structured into teams to develop and implement a data science project. These initiatives will be RWD projects that will address current healthcare problems. Completion of projects will be a form of learning into practice and will establish a foundation for further commercial activity or research. The proposed blended format will prepare future (postgraduate students) and current healthcare professionals with the required skills and knowledge to conduct and be part of RWE projects and will help develop them to the growing need of data-analysis skills in the healthcare field. Topics and key concepts will include principles of RWE in health, information governance for RWE, design, methodology and framework for RWE, and RWE exploratory analysis and evaluation.

One of the primary learning outcomes of the course will be the ability to apply an established RWE framework that ensures transparency and integrity around the collection, analysis and use of RWD. Other learning outcomes include: (1) to provide learners with formal knowledge of RWE, (2) to provide learners with experiences that expand on their existing knowledge of RWE, (3) to have learners determine how to learn about the evolving topic, (4) to have learners self-organise into social networks to develop knowledge and address situated problems, (5) to have learners collaborate to master the competencies required to solve RWD challenges. Each learning outcome will be delivered on a module basis.

### Production

A pan-European collaboration has been established including Université Grenoble Alpes, Karolinska Institutet, Imperial College London and the University of Oxford. Video presentations will be made with partners and recorded and edited by the production team. Written pieces such as articles, case studies PowerPoint presentations, quizzes and assessment will be developed by the production team and partners prior to integration onto the delivery platform. The face-to-face on-campus course will be developed and offered at the Medical Sciences Division at the University of Oxford. To evaluate the content before it is released, we will run a beta trial of MOOC with a sample of target learners.

### Learner recruitment

We will develop a marketing campaign focused on social media and online advertising in order to recruit learners; also coordinating with the marketing of other courses. We will also cross-market to our database of 1000+ learners. Specifically, course promotion will involve a variety of methods to include (1) the institution’s own website; (2) social media (eg, Facebook, Twitter, Google+, LinkedIn); (3) posting of articles via distance learning portals; (4) materials such as flyers, brochures, roll-ups and posters; (5) emails; (6) regular course newsletters. Providing the recruiting process on public platforms will ensure the process is clear, open and transparent. As a key element of the course will be connectivity between participants, learners will be encouraged to share knowledge, experience and ideas throughout the course by discussion forums and via social media. We will continue the same promotional activities during the course delivery and to actively encourage partition, newsletter and course social media hashtag will be available during and 1 year is the course.

### Problems anticipated

To create a feasible work plan, potential risks and contingencies were identified in the following areas:

Instructional design:

Key risks: On-boarding of new staff.
Contingency: Imperial College is contributing existing staff familiar with this form of course design.

Learning management system design:

Key risks: Requesting functionality not possible from the learning management system.
Contingency: Will mitigate risk through early engagement with faculty and planning in advance.

The beta trial of course:

Key risks: Non-availability of test students.
Contingency: Will incentivise beta participant learners.

Live implementation of course:

Key risks: Student recruitment and marketing.
Contingency: Will market the course from the beginning of the year to ensure high student enrolment numbers.

Evaluation:

Key risks: Ethical considerations in the evaluation.
Contingency: Will seek ethical approval of the evaluation process.

### MOOC outcomes

Success for this course will be maintaining an active cohort of learners who will engage in continued discussion on these topics (eg, via social media), where we also establish a basis for possible collaborations on future initiatives. The short-term impact of the course includes enabling participants to join networks of data science in healthcare and engaging and educating participants on the basic skills needed to conduct RWE projects in their work or studies. The long-term impact includes enabling participants to maintain their presence in networks of data science in healthcare and enabling participants to conduct RWE projects in their work and contribute to the improvement of healthcare through the use of data and evidence-based approaches.

We expect learners to take skills taught in MOOC and use them to seek new employment or start to initiatives in these domains. Learners will be able to start new RWE initiatives as a result of the practical RWE projects they will complete in MOOC. Learners will submit a project using an RWE framework taught in MOOC that will be peer assessed. To make sure that projects are assessed properly, a rubric with clearly defined guidelines and reviewing criteria will be provided. The course coordinator will also facilitate these exercises. The successful completion of the RWE project (both submitting a project and peer assessing the project) will give learners the basic skills to carry out an RWE project.

### Evaluation

MOOC evaluation is a significant practice to assess its value and effectiveness.^16^ To evaluate the course’s impact, we will complete an evaluation on a participant level to measure the MOOC training effects over me. We plan to prepare the evaluation findings in the form of a report that can be shared with our partners. To ensure evaluation results are shared and used for the improvement of future projects, a report summarising the research findings of our MOOC evaluation will be published in a peer-reviewed journal. Findings shall also be disseminated at an international conference.

During the course launch, we will use the Experience API (xAPI) to track all learning activities taking place in the course. This software tracks and records all learning activities with the learning management system and will be used to assist to provide real-time feedback to the course organisers on how learners are responding to the course. Moreover, pre-MOOC and post-MOOC survey data will be captured to evaluate learner’s satisfaction with the course, and whether it met their expectations. Course metrics such as retention on the course, number of participants in discussion posts, number of participants in the course’s social media posts and the number of learners who completed the RWE projects will all be used as indicators for course impact.

Other postproject monitoring will be conducted with learners from MOOC via two interviews, the first 1 month after the course, the second, 3 months postcourse. We are aiming to recruit 16 participants for interviews and if more than 16 agree to an interview, we will include participants who represent as wide a range of data science backgrounds and levels of MOOC completion as possible. We have targeted 16 learners because based on the literature,^17^ a study size of 16 is appropriate for collection of qualitative data and for providing sufficient insight into the questions studied. However, should there be a need for further exploration of phenomena under study (and themes and findings which emerge), we will attempt to continue interviewing participants until saturation is reached on the most important themes relating primarily to the course’s effectiveness.

A mixed-methods approach will be used to analyse these outcomes, incorporating precourse and postcourse survey results, semistructured interviews of a subset of the learners and social media network analysis. The evaluation’s main focus will be learners’ application of the practical skills gained through MOOC in their workplace using Kirkpatrick evaluation method and will be conducted on selected learners a year postimplementation.^18^ The Kirkpatrick evaluation is a methodology that evaluates the success of training courses, and it is a suitable choice for evaluating the success of the proposed MOOC since MOOC’s aim is to teach hands-on skills in RWE data analysis.^18^ In addition, course metrics such as retention on the course, number of learners who completed the RWE projects will all be used as indicators of course outcomes achievement.

### Patient and public involvement

Members of the public were informed the development of research questions and study objectives via a workshop held at the European Scientific Institute in July 2017.

### Sustainability

We plan to sustain the course and course updates via internal funding following this investment round and sustaining the course through course certification fees.

## Ethics and dissemination

Ethics approval for this study was obtained from Imperial College London through the Education Ethics Review Process (EERP) (EERP1617-030). A report summarising the research findings will be published in a peer-reviewed journal. A presentation will be given to a selected audience of health professionals and academics, to include individuals from Imperial College. Findings will also be presented at an international conference.

## Supplementary Material

Reviewer comments

Author's manuscript
