# Supplementary material for: Real-world evidence for postgraduate students and professionals in healthcare: protocol for the design of a blended massive open online course
Source: BMJ Open. 2018 Oct 4;8(9):e025196. doi: 10.1136/bmjopen-2018-025196 (PMC6173253; doi:10.1136/bmjopen-2018-025196)
Supplement: Author's manuscript [file bmjopen-2018-025196.draft_revisions.pdf]

BMJ Open is committed to open peer review. As part of this commitment we make the peer review history of every article we publish publicly available.

When an article is published we post the peer reviewers' comments and the authors' responses online. We also post the versions of the paper that were used during peer review. These are the versions that the peer review comments apply to.

The versions of the paper that follow are the versions that were submitted during the peer review process. They are not the versions of record or the final published versions. They should not be cited or distributed as the published version of this manuscript.

BMJ Open is an open access journal and the full, final, typeset and author-corrected version of record of the manuscript is available on our site with no access controls, subscription charges or pay-per-view fees (http://bmjopen.bmj.com).

If you have any questions on BMJ Open's open peer review process please email info.bmjopen@bmj.com

BMJ Open

Real World Evidence for postgraduate students and professionals in healthcare: protocol for the design of a blended Massive Open Online Course

Journal:	*BMJ Open*
Manuscript ID	bmjopen-2018-025196
Article Type:	Protocol
Date Submitted by the Author:	03-Jul-2018

Complete List of Authors:	Meinert, Edward; Imperial College London, Primary Care and Public Health; University of Oxford, Paediatrics<br>Alturkistani, Abrar; Imperial College London, Primary Care and Public Health<br>Car, Josip; Imperial College London, Primary Care and Social Medicine<br>Carter, Alison; University of Oxford, Paediatrics<br>Wells, Glenn; Oxford Academic Health Science Centre<br>Brindley, David; University of Oxford, Paediatrics; University of Oxford, Said Buisness School

Keywords:	Massive Open Online Course, Real World Evidence, Real World Data, EDUCATION & TRAINING (see Medical Education & Training), Data science

SCHOLARONE™
Manuscripts

For peer review only

Real World Evidence for postgraduate students and professionals in healthcare: protocol for the design of a blended Massive Open Online Course

Edward Meinert, Abrar Alturkistani, Josip Car, Alison Carter, Glenn Wells, David Brindley

Department of Paediatrics, University of Oxford, OX3 9DU Oxford, United Kingdom Edward Meinert Sir David Cooksey Fellow in Healthcare Translation Global eHealth Unit, Department of Public Health and Primary Care, School of Public Health, Imperial College London, W6 8RP London, United Kingdom Edward Meinert Honorary Research Fellow Department of Infectious Disease Epidemiology, Faculty of Medicine, School of Public Health, Imperial College London, W6 8RP London, United Kingdom Abrar Alturkistani Research Assistant Global eHealth Unit, Department of Public Health and Primary Care, School of Public Health, Imperial College London, W6 8RP London, United Kingdom Josip Car Director of the Global eHealth Unit Department of Paediatrics, University of Oxford, OX3 9DU Oxford, United Kingdom Alison Carter Research Associate Oxford Academic Health Science Centre, OX4 4GA Oxford, United Kingdom Glenn Wells Chief Operating Officer Department of Paediatrics, University of Oxford, OX3 9DU Oxford, United Kingdom David Brindley Senior Research Fellow

Correspondence to: Edward Meinert edward.meinert@paediatrics.ox.ac.uk, e.meinert14@imperial.ac.uk 00447824446808

Word Count: 3476

Keywords: Massive Open Online Course; Real World Evidence; Information Science; Continuing Education

For peer review only

ABSTRACT

Introduction

There is an increased need for improving data science skills of healthcare professionals. Massive Open Online Courses (MOOCs) provide the opportunity to train professionals in a sustainable and cost-effective way. We present a protocol for the design and development of a blended MOOC on RWE aimed at improving RWE data science skills. The primary objective is to provide the opportunity to understand the fundamentals of RWE data science and to implement methods for analysing RWD. The blended format of the MOOC will combine the expertise of healthcare professionals joining the course online with the students on-campus. We expect learners to take skills taught in the MOOC and use them to seek new employment or start to initiatives in these domains.

Methods and Analysis

The proposed MOOC will be developed through a blended format using the ADDIE (Analysis, Design, Development, Implementation and Evaluation) instructional design model and following the connectivist-heutagogical learning theories (as a hybrid MOOC). The target learners will include postgraduate students and professionals working in the health-related roles with interest in data science. An evaluation of the MOOC will be performed to assess the MOOCs success in meeting its intended outcomes and to improve future iterations of the course.

Ethics and dissemination

The education course design protocol was approved by EIT Health (Grant 18654) as part of the EIT Health CAMPUS Deferred Call for Innovative Education 2018. Results will be published in a peer-reviewed journal.

For peer review only

STRENGTHS AND LIMITATIONS OF THIS STUDY

- The strengths of the blended massive open online course design is the use of pedagogical design based on the connectivist and heutagogical learning theories
- Another strength is that the evaluation study of the course is embedded in the course design increasing the likelihood of a timely evaluation
- Successful implementation and evaluation of the course will depend on learner recruitment and retention

INTRODUCTION

Healthcare is becoming increasingly dependent on data analytics for improvement of care, efficiency, and quality, and getting more value from resources.[1] However, the fast-growing data analysis field requires constant and up-to-date training in new skills.[2] There is a high demand for trained data scientists in healthcare,[1] as health is associated with challenges that can solved by advanced data science methods with the potential to improve delivery of patient outcomes by increasing efficiency and effectiveness. For example, using big data methods on the vast quantity of Real World Data (RWD), enables providers the ability to create predictive models which can help accurately identify risk of disease and improved and personalized preventive care for patients.[3] RWD includes the vast quantity of data that falls outside the boundaries of controlled clinical trials, which can be utilised for: measuring healthcare resource-use and the burden of disease, evaluating prescribing patterns or clinical outcomes of a new medicine, describing current treatment patterns for a patient group for baseline information.[4] Real World Evidence (RWE), which is derived as a result of RWD, is starting to play a significant role in decision making and it is expected to grow in the future.[3]

Mentoring, team-based training and self-study are recognised as effective measures to train professionals in data analytical skills.[2] Massive Open Online Courses (MOOCs), a form of web-based online learning, provide skills adoption opportunities through the provision of learning in an open, publicly available platform that is often free of charge on specialised topics.[5] MOOCs can increase the knowledge of professionals in a new topic,[6] and can help them gain new skills.[7] MOOCs have gained large popularity in different fields in the last ten years,[8] but despite the availability of numerous MOOCs on data science, currently, there are very limited courses focusing on the topic of RWE. Also, there has been limited, but increasing use of MOOCs in a blended capacity and use of this project's proposed instructional design shall create the opportunity to observe how a MOOC can be managed in this capacity, considering the logic and structural issues incumbent in this format.[9]

Dealing with RWD can be daunting and challenging due to its volume, scale and the requirement to understand the source data and its collection in context. Researchers and healthcare professionals that want to utilise RWD need to learn new methodologies and frameworks which deviate from standard research methods. Although MOOCs provide an opportunity to train healthcare professional in data science skills, very limited number of MOOCs have touched on the topic of RWE. This project will address this by creating an educational program that focuses on RWD data analysis methods and skills to improve healthcare delivery.

For peer review only

The proposed blended-MOOC will prepare future (postgraduate students) and current healthcare professionals with the required skills and knowledge to conduct and be part of RWE projects. Through the course, we aim to impact the learners' knowledge, skills and attitudes on the use of RWE data science in healthcare, as well as their communication and participation in networks of data science in healthcare. We also aim to inspire the use of RWE methods across various postgraduate curriculum disciplines, National Health Service (NHS) commissioning support organisations, healthcare regulation organisations and life sciences industries (i.e. pharmaceuticals, biotechnology and medical devices). RWE MOOC was funded in April 2018. Planning preparation and planning for the course will begin in June 2018 with the identification of key themes and concepts to be translated into content. Course production will commence in June 2018 and the course will go live in December 2018. The course will be deployed on the Innoenergy X-KIC FutureLearn Platform.

We aim to bring the online learning component (MOOC) together with a face-to-face university level course, to enhance the learning experience and to challenge and enrich postgraduate level course taught with traditional methods. This will allow diversifying knowledge delivery mediums and will enable university students to be exposed to a global community of learners. The MOOC will allow learners the flexibility of amending the learning from any location, the advantage of meeting a large number of students outside their regular university classrooms and will meet EIT Health's call for "Massive dissemination" of innovative education ideas. A blended course will allow knowledge integration between the university students and the global learners joining the MOOC from all over the world, which can add richness and enhance the learning experience for all learners. The limited availability of courses on RWE, a topic that is gaining popularity in healthcare and the use of the latest advances in online education (blended MOOC) is what makes our proposed programme unique regarding the topic and educational strategies used.

METHODS AND ANALYSIS

MOOC Overview

The project will deliver an education programme via a MOOC and a face to face course, to create a blended format for postgraduate students and professionals interested in the application of RWE data analysis and in furthering their knowledge base and skill set to include conducting and commissioning RWD analysis. To help meet this goal, a key objective of the MOOC is to establish a global network of people to continue and advance the dialogue on data science in healthcare. It is expected that the MOOC will provide the opportunity to understand the fundamentals of RWE data science and to implement methods for analysing RWD.

MOOC development

The course will be mainly organized from Imperial College's Global eHealth Unit and course material will be filmed on Imperial College premises. The face-to-face component of the course will be offered at the University of Oxford. The course will be delivered in five weeks (5 modules). However, the fifth module will only be offered to learners who sign up for certification. The course will be offered on specific dates and will be run for 5 weeks, and each module will be released on a

For peer review only - http://bmjopen.bmj.com/site/about/guidelines.xhtml

weekly basis. Pilot implementation of the course will occur in 2018; partners will construct the core course programme, developing the face-to-face course and the complementary digital learning. Two face-to-face workshops will be held in the UK to pilot the blended course design. Scale-out will occur in 2019, face-to-face workshops will be held in France, Sweden, Spain and the UK, one per quarter. This will expand the reach of the programme and facilitate project development which will be done in conjunction with industry partners in each EU region. Workshops will include industry partners focused on the design, development and implementation of RWE methods

MOOC Pedagogical design

Course design will be based on the hybrid connectivist-heutagogical learning theories. The connectivism theory emphasizes the use of networking and communication to deliver the learning material, where interactions and discussions between learners can also contribute to the introduction of new knowledge [10]. Heutagogy principles emphasizes creating competent learners through touching on "learner autonomy and self-directedness."[11] To enhance the connectivist characteristics of course, the digital platform will be created in a way that can enable learners to create social networks and communicate with like-minded individuals. One of the objectives of the course is to establish a global network to continue and advance the dialogue on data science in healthcare. Attendance in a course with healthcare professionals from around the world will give the postgraduate students the opportunity to appreciate the role of data in real life clinical settings and introduce them to methods and challenges faced in practical healthcare settings. At the same time, healthcare professionals joining the course will be able to communicate and engage with other professionals who will be possible using data in a different healthcare setting; clinical or non-clinical. The heutagogical characteristics of the course will be enhanced, by allowing learners the flexibility to go through the course material in their own time and to move through the material according to their preference. This learning principle will also be enhanced by incorporating social media into the discussions, [11] and creating a course hashtag that can be accessed by learners for further discussion.

Teaching strategies

The course plan will be prepared following the ADDIE model (instructional design model) consisting of Analysis, Design, Development, Implementation and Evaluation.[12] The ADDIE model provides a systematic, step-by-step framework to ensure the course is developed in a structured and a comprehensive way.[13] To teach innovation, learners will be structured into teams to develop and implement a data science project. These initiatives will be RWE projects that will address current health problems. Our assessment strategies will include both formative (polls and peer-assessment) and summative assessments (multiple choice questions/module). We will offer an optional fee-based certificate for completion of the MOOC and a separate project. The project will involve the proposal of an RWE data analysis methodology (using techniques learned in the course) to solving a current healthcare problem. Learners will be engaged in collaborative exercises and reflective discussions with other learners where the 'teacher' will act as a facilitator and a contributor to the discussions. Each module will provide a list of required reading, recommended reading, and RWE related datasets and tools to allow learners to individualise their learning and explore other sources of knowledge.[14]

For peer review only - http://bmjopen.bmj.com/site/about/guidelines.xhtml

Target Learners

The course will cater to two types of learners: current university level students (postgraduate) and professionals in the healthcare field (e.g. professionals working in healthcare, commissioners, policy makers, healthcare regulators and those working in the life sciences.); (1) who have some background knowledge of statistics in healthcare and/or (2) who carry out hands-on data analysis in their work or studies. The target audience will include postgraduate students in health-related fields with an interest in the use of RWD and RWE data science in healthcare and professionals working in NHS commissioning, healthcare regulation and life sciences. Selection parameters will be based on participant's occupation and statistical knowledge. Participants who are postgraduate students or professionals in healthcare fields, who have completed a basic university-level statistics course or who have professional experience in data analysis and research will be eligible to join the MOOC. The required prerequisite is knowledge in basic statistics and understanding of statistical measures used in healthcare, which is met if the learner completed a university-level basic statistics course or professionally works on data analysis.

Learning outcomes

The content from the course develops skills in the context of RWE, including frameworks for analysing and evaluating content (data) and subsequently, will encourage them to embark on healthcare RWE related projects, innovation and entrepreneurship. To complete the course, learners will be structured into teams to develop and implement a data science project. These initiatives will be RWD projects that will address current healthcare problems. Completion of projects will be a form of learning into practice and establish a foundation for further commercial activity or research. The proposed blended format will prepare future (postgraduate students) and current healthcare professionals with the required skills and knowledge to conduct and be part of RWE projects and will help develop them to the growing need of data analysis skills in the healthcare field. Topics and key concepts will include Principles of RWE in Health, Information Governance for RWE, Design, Methodology and Framework for RWE, and RWE Exploratory Analysis and Evaluation.

One of the primary learning outcomes of the course will be the ability to apply an established RWE framework that ensures transparency and integrity around the collection, analysis and use of RWD. Other learning outcomes include: (1) To provide learners with formal knowledge of RWE, (2) To provide learners with experiences that expand upon their existing knowledge of RWE, (3) To have learners determine how to learn about the evolving topic, (4) To have learners self-organise into social networks to develop knowledge and address situated problems, (5) To have learners collaborate to master the competencies required to solve RWD challenges. Each learning outcome will be delivered on a module basis.

Production

A pan-European collaboration has been established including Université Grenoble Alpes, Karolinska Institutet, Imperial College London and the University of Oxford. Video presentations will be made with partners and recorded and edited by the production team. Written pieces such as articles, case studies powerpoint presentations, quizzes and assessment will be developed by the production

For peer review only - http://bmjopen.bmj.com/site/about/guidelines.xhtml

team and partners prior to integration onto the delivery platform. The face-to-face, the on-campus course will be developed and offered at the Medical Sciences Division at the University of Oxford. To evaluate the content before it is released we will run a beta trial of the MOOC with a sample of target learners.

Learner Recruitment

We will develop a marketing campaign focused on social media and online advertising in order to recruit learners; also coordinating with the marketing of other courses. We will also cross market to our database of 1000+ learners. Specifically, course promotion will involve a variety of methods to include (1) the Institution's own website (2) social media (e.g. Facebook, Twitter, Google+, LinkedIn) (3) posting of articles via Distance Learning Portals (4) materials such as flyers, brochures, roll-ups and posters (5) email mailings (6) regular course newsletters. Providing the recruiting process on public platforms will ensure the process is clear, open and transparent. As a key element of the course will be connectivity between participants, learners will be encouraged to share knowledge, experience and ideas throughout the course by discussion forums and via social media. We will continue the same promotion activities during the course delivery and to actively encourage partition, newsletter, and course social media hashtag will be available during and one year is the course.

Problems anticipated

To create a feasible work plan, potential risks and contingencies were identified in the following areas:

Instructional design:

Key risks:

on-boarding of new staff

Contingency:

Imperial College is contributing existing staff familiar with this form of course design.

Learning management system design:

Key risks:

Requesting functionality not possible from the LMS

Contingency:

Will mitigate risk through early engagement with faculty and planning in advance.

The beta trial of course:

Key risks:

Non-availability of test students

Contingency:

Will incentive beta participant learners

Live implementation of course:

Key risks:

Student recruitment and marketing

Contingency:

Will market the course from the beginning of the year to ensure high student enrolment numbers

For peer review only - http://bmjopen.bmj.com/site/about/guidelines.xhtml

Evaluation:

Key risks:

Ethical considerations in the evaluation

Contingency:

Will seek ethical approval of the evaluation process

MOOC Outcomes

Success for this course will be maintaining an active cohort of learners who will engage in continued discussion on these topics (e.g. via social media), where we also establish a basis for possible collaborations on future initiatives. The short-term impact of the course includes enabling participants to join networks of data science in healthcare and engaging and educating participants on the basic skills needed to conduct RWE projects in their work or studies. The long-term impact includes enabling participants to maintain their presence in networks of data science in healthcare and enabling participants to conduct RWE projects in their work and contribute to the improvement of healthcare through the use of data and evidence-based approaches.

We expect learners to take skills taught in the MOOC and use them to seek new employment or start to initiatives in these domains. Learners will be able to start new RWE initiatives as a result of the practical RWE projects they will complete in the MOOC. Learners will submit a project using an RWE framework taught in the MOOC that will be peer-assessed. To make sure that projects are assessed properly, a rubric with clearly defined guidelines and reviewing criteria will be provided. The course coordinator will also facilitate these exercises. The successful completion of the RWE project (both submitting a project and peer assessing the project) will give learners the basic skills to carry out an RWE project.

Evaluation

MOOC evaluation is a significant practice to assess its value and effectiveness. [15] To evaluate the course's impact, we will complete an evaluation on a participant-level to measure the continuation of MOOC effects over me. We plan to prepare the evaluation findings in the form of a report that can be shared with our partners. The evaluation will be mainly used to evaluate the impact of the current project learners became involved in and its success in delivering the intended outcomes. However, the evaluation will also provide insights that can be learned to enhance future iterations of the MOOC. To ensure evaluation results are shared and used for the improvement of future projects, a report summarising the research findings of our MOOC evaluation will be published in a peer-reviewed journal. Findings shall also be disseminated at an international conference.

During the course launch, we will use xAPI to track all learning activity taking place in the course. This software tracks and records all learning activity with the learning management system and will be used to assist to provide real-me feedback to the course organisers on how learners are responding to the course. Moreover, pre-MOOC and post-MOOC survey data will be captured to evaluate learner's satisfaction with the course, and whether or not it met their expectations. Course metrics such as retention on the course, number of participants in discussion posts, number of participants in the course's social media posts and the number of learners who completed the RWE projects will all be used as indicators for course impact.

For peer review only - http://bmjopen.bmj.com/site/about/guidelines.xhtml

Other post-project monitoring will be conducted with learners from the MOOC via 2 interviews, the first 1 month are the course, the second, 3 months post-course. We are aiming to recruit 16 participants for interviews and if more than 16 agree to an interview, we will include participants who represent as wide a range of data science backgrounds and levels of MOOC completion as possible (thereby liming demographic duplication and the possible number of individuals to interview). We have targeted 16 learners because based on the literature, [16] a study size of 16 is appropriate for collection of qualitative data and for providing sufficient insight into the questions studied. However, should there be a need for further exploration of phenomena under study (and themes and findings which emerge), we will attempt to continue interviewing participants until saturation is reached on the most important themes relating primarily to the course's effectiveness

A mixed methods approach will be used to analyse these outcomes, incorporating pre-course and post-course survey results, semi-structured interviews of a subset of the learners and social media network analysis. The evaluations main focus will be learners' application of the practical skills gained through the MOOC in their workplace using Kirkpatrick evaluation method and will be conducted on selected learners a year post-implementation.[17] The Kirkpatrick evaluation is a methodology that evaluates the success of training courses, and it is a suitable choice for evaluating the success of the proposed MOOC since the MOOC's aim is to teach hands-on skills in RWE data analysis.[17] In addition, course metrics such as retention on the course, number of learners who completed the RWE projects will all be used as indicators of course outcomes achievement.

Sustainability

We plan to sustain the course and course updates via internal funding following this investment round and sustaining the course through course certification fees.

ETHICS AND DISSEMINATION

The protocol for the course was approved by EIT Health (Grant 18654) as part of the EIT Health CAMPUS Deferred Call for Innovative Education 2018. Results will be published in a peer-reviewed journal.

Contributors

EM conceived the study and drafted the manuscript. JC contributed to the protocol design. All authors read and approved the final manuscript. EM is the guarantor.

Acknowledgements

This work was funded by EIT Health (Grant 18654). This work was also supported by the Sir David Cooksey Fellowship in Healthcare Translation and the SENS Research Foundation.

For peer review only - http://bmjopen.bmj.com/site/about/guidelines.xhtml

Competing interests

The ICMJE uniform disclosure form at http://www.icmje.org/conflicts-of-interest/ was completed by all authors. All authors declare that they have no relevant conflicts of interest, no financial relationships that could potentially influence the submitted manuscript. All authors do not have any patents that may be relevant to the manuscript or any relationship, circumstances or conditions that may lead to conflicts of interest. This article represents the authors' individual opinions and may not necessarily represent the viewpoints of their employers. D.B. is a stockholder in Translation Ventures. Ltd. (Charlbury, Oxfordshire, UK) and IP Asset Ventures Ltd. (Oxford, Oxfordshire, UK), companies that, among other services, provide cell therapy biomanufacturing, regulatory, and financial advice to pharmaceutical clients. D.B. is also subject to the CFS institute's codes, standards, and guidelines, so he must stress that this piece is provided for academic interest only and must not be seen in any way as an investment recommendation.

Funding statement

This work was funded by EIT Health (Grant 18654).

For peer review only

REFERENCES

1. Wang Y, Kung L, Byrd TA. Big data analytics: Understanding its capabilities and potential benefits for healthcare organizations. *Technological Forecasting and Social Change* 2018;**126**:3–13. doi:10.1016/j.techfore.2015.12.019
2. American Management Association (AMA). Conquering Big Data: Building Analytical Skills in Your Organization. American Management Association Press 2013. https://cdns3.trainingindustry.com/media/16687264/conquering_big_data_survey_final.pdf (accessed 2 Jul 2018).
3. Sherman RE, Anderson SA, Dal Pan GJ, *et al.* Real-world evidence—what is it and what can it tell us. *N Engl J Med* 2016;**375**:2293–2297.
4. Hubbard TE, Paradis R, The Network for Excellence in Health Care Innovation. The Network for Excellence in Health Innovation: Issue Breif: Real World Evidence: A New Era for Health Care Innovation. One Broadway, Cambridge, MA 02142: : The Network for Excellence in Health Innovation 2015. https://www.nehi.net/writable/publication_files/file/rwe_issue_brief_final.pdf (accessed 2 Jul 2018).
5. Steels L. The Coming of MOOCs. In: *Volume 6: Music Learning with Massive Open Online Courses (MOOCs)*. IOS Press 3–20.
6. Hossain MS, Shofiqul Islam M, Glinsky JV, *et al.* A massive open online course (MOOC) can be used to teach physiotherapy students about spinal cord injuries: a randomised trial. *J Physiother* 2015;**61**:21–7. doi:10.1016/j.jphys.2014.09.008
7. George PP, Papachristou N, Belisario JM, *et al.* Online eLearning for undergraduates in health professions: A systematic review of the impact on knowledge, skills, attitudes and satisfaction. *J Glob Health* 2014;**4**:010406. doi:10.7189/jogh.04.010406
8. Barnes C. MOOCs: The Challenges for Academic Librarians. *Australian Academic & Research Libraries* 2013;**44**:163–75. doi:10.1080/00048623.2013.821048
9. Israel MJ. Effectiveness of Integrating MOOCs in Traditional Classrooms for Undergraduate Students. *The International Review of Research in Open and Distributed Learning* 2015;**16**. doi:10.19173/irrodl.v16i5.2222
10. Duke B, Harper G, Johnston M. Connectivism as a digital age learning theory. *The International HETL Review* 2013;**2013**:4–13.
11. Blaschke LM. Heutagogy and lifelong learning: A review of heutagogical practice and self-determined learning. *The International Review of Research in Open and Distributed Learning* 2012;**13**:56–71.
12. Kruse K. An Introduction to the ADDIE Model for Instructional Designers. E-Learning Heroes. 2002.https://community.articulate.com/articles/an-introduction-to-the-addie-model-for-instructional-designers (accessed 2 Jul 2018).

For peer review only - http://bmjopen.bmj.com/site/about/guidelines.xhtml

13. Peterson C. Bringing ADDIE to Life: Instructional Design at Its Best. *Journal of Educational Multimedia and Hypermedia* 2003;**12**:227–41.
14. Manallack DT, Yuriev E. Ten Simple Rules for Developing a MOOC. *PLOS Computational Biology* 2016;**12**:e1005061. doi:10.1371/journal.pcbi.1005061
15. Chapman SA, Goodman S, Jawitz J, *et al.* A strategy for monitoring and evaluating massive open online courses. *Evaluation and Program Planning* 2016;**57**:55–63. doi:10.1016/j.evalprogplan.2016.04.006
16. Adler P, Adler P. Expert voices. In Baker SE, Edwards R, Doidge M. How many qualitative interviews is enough?: Expert voices and early career reflections on sampling and cases in qualitative research. 2012.
17. Kirkpatrick DL. *Evaluating Training Programs: The Four Levels*. ReadHowYouWant.com 2009.

For peer review only

For peer review only - http://bmjopen.bmj.com/site/about/guidelines.xhtml

BMJ Open

Real World Evidence for postgraduate students and professionals in healthcare: protocol for the design of a blended Massive Open Online Course

Journal:	*BMJ Open*
Manuscript ID	bmjopen-2018-025196.R1
Article Type:	Protocol
Date Submitted by the Author:	10-Jul-2018
Complete List of Authors:	Meinert, Edward; Imperial College London, Primary Care and Public Health; University of Oxford, Paediatrics<br>Alturkistani, Abrar; Imperial College London, Primary Care and Public Health<br>Car, Josip; Imperial College London, Primary Care and Social Medicine<br>Carter, Alison; University of Oxford, Paediatrics<br>Wells, Glenn; Oxford Academic Health Science Centre<br>Brindley, David; University of Oxford, Paediatrics; University of Oxford, Said Buisness School
**Primary Subject Heading**:	Medical education and training
Secondary Subject Heading:	Evidence based practice, Health services research, Public health, Qualitative research
Keywords:	Massive Open Online Course, Real World Evidence, Real World Data, EDUCATION & TRAINING (see Medical Education & Training), Data science

SCHOLARONE™
Manuscripts

Real World Evidence for postgraduate students and professionals in healthcare: protocol for the design of a blended Massive Open Online Course

Edward Meinert, Abrar Alturkistani, Josip Car, Alison Carter, Glenn Wells, David Brindley

Department of Paediatrics, University of Oxford, OX3 9DU Oxford, United Kingdom Edward Meinert Sir David Cooksey Fellow in Healthcare Translation Global eHealth Unit, Department of Public Health and Primary Care, School of Public Health, Imperial College London, W6 8RP London, United Kingdom Edward Meinert Honorary Research Fellow Department of Infectious Disease Epidemiology, Faculty of Medicine, School of Public Health, Imperial College London, W6 8RP London, United Kingdom Abrar Alturkistani Research Assistant Global eHealth Unit, Department of Public Health and Primary Care, School of Public Health, Imperial College London, W6 8RP London, United Kingdom Josip Car Director of the Global eHealth Unit Department of Paediatrics, University of Oxford, OX3 9DU Oxford, United Kingdom Alison Carter Research Associate Oxford Academic Health Science Centre, OX4 4GA Oxford, United Kingdom Glenn Wells Chief Operating Officer Department of Paediatrics, University of Oxford, OX3 9DU Oxford, United Kingdom David Brindley Senior Research Fellow

Correspondence to: Edward Meinert edward.meinert@paediatrics.ox.ac.uk, e.meinert14@imperial.ac.uk 00447824446808

Word Count: 3476

Keywords: Massive Open Online Course; Real World Evidence; Information Science; Continuing Education

For peer review only

ABSTRACT

Introduction

There is an increased need for improving data science skills of healthcare professionals. Massive Open Online Courses (MOOCs) provide the opportunity to train professionals in a sustainable and cost-effective way. We present a protocol for the design and development of a blended MOOC on RWE aimed at improving RWE data science skills. The primary objective is to provide the opportunity to understand the fundamentals of RWE data science and to implement methods for analysing RWD. The blended format of the MOOC will combine the expertise of healthcare professionals joining the course online with the students on-campus. We expect learners to take skills taught in the MOOC and use them to seek new employment or start to initiatives in these domains.

Methods and Analysis

The proposed MOOC will be developed through a blended format using the ADDIE (Analysis, Design, Development, Implementation and Evaluation) instructional design model and following the connectivist-heutagogical learning theories (as a hybrid MOOC). The target learners will include postgraduate students and professionals working in the health-related roles with interest in data science. An evaluation of the MOOC will be performed to assess the MOOCs success in meeting its intended outcomes and to improve future iterations of the course.

Ethics and dissemination

The education course design protocol was approved by EIT Health (Grant 18654) as part of the EIT Health CAMPUS Deferred Call for Innovative Education 2018. Results will be published in a peer-reviewed journal.

For peer review only - http://bmjopen.bmj.com/site/about/guidelines.xhtml

STRENGTHS AND LIMITATIONS OF THIS STUDY

- The strengths of the blended massive open online course design is the use of pedagogical design based on the connectivist and heutagogical learning theories
- Another strength is that the evaluation study of the course is embedded in the course design increasing the likelihood of a timely evaluation
- Successful implementation and evaluation of the course will depend on learner recruitment and retention

INTRODUCTION

Healthcare is becoming increasingly dependent on data analytics for improvement of care, efficiency, and quality, and getting more value from resources.[1] However, the fast-growing data analysis field requires constant and up-to-date training in new skills.[2] There is a high demand for trained data scientists in healthcare,[1] as health is associated with challenges that can solved by advanced data science methods with the potential to improve delivery of patient outcomes by increasing efficiency and effectiveness. For example, using big data methods on the vast quantity of Real World Data (RWD), enables providers the ability to create predictive models which can help accurately identify risk of disease and improved and personalized preventive care for patients.[3] RWD includes the vast quantity of data that falls outside the boundaries of controlled clinical trials, which can be utilised for: measuring healthcare resource-use and the burden of disease, evaluating prescribing patterns or clinical outcomes of a new medicine, describing current treatment patterns for a patient group for baseline information.[4] Real World Evidence (RWE), which is derived as a result of RWD, is starting to play a significant role in decision making and it is expected to grow in the future.[3]

Mentoring, team-based training and self-study are recognised as effective measures to train professionals in data analytical skills.[2] Massive Open Online Courses (MOOCs), a form of web-based online learning, provide skills adoption opportunities through the provision of learning in an open, publicly available platform that is often free of charge on specialised topics.[5] MOOCs can increase the knowledge of professionals in a new topic,[6] and can help them gain new skills.[7] MOOCs have gained large popularity in different fields in the last ten years,[8] but despite the availability of numerous MOOCs on data science, currently, there are very limited courses focusing on the topic of RWE. Also, there has been limited, but increasing use of MOOCs in a blended capacity and use of this project's proposed instructional design shall create the opportunity to observe how a MOOC can be managed in this capacity, considering the logic and structural issues incumbent in this format.[9]

Dealing with RWD can be daunting and challenging due to its volume, scale and the requirement to understand the source data and its collection in context. Researchers and healthcare professionals that want to utilise RWD need to learn new methodologies and frameworks which deviate from standard research methods. Although MOOCs provide an opportunity to train healthcare professional in data science skills, very limited number of MOOCs have touched on the topic of RWE. This project will address this by creating an educational program that focuses on RWD data analysis methods and skills to improve healthcare delivery.

For peer review only - http://bmjopen.bmj.com/site/about/guidelines.xhtml

The proposed blended-MOOC will prepare future (postgraduate students) and current healthcare professionals with the required skills and knowledge to conduct and be part of RWE projects. Through the course, we aim to impact the learners' knowledge, skills and attitudes on the use of RWE data science in healthcare, as well as their communication and participation in networks of data science in healthcare. We also aim to inspire the use of RWE methods across various postgraduate curriculum disciplines, National Health Service (NHS) commissioning support organisations, healthcare regulation organisations and life sciences industries (i.e. pharmaceuticals, biotechnology and medical devices). RWE MOOC was funded in April 2018. Planning preparation and planning for the course will begin in June 2018 with the identification of key themes and concepts to be translated into content. Course production will commence in June 2018 and the course will go live in December 2018. The course will be deployed on the Innoenergy X-KIC FutureLearn Platform. The impact and efficacy of this delivery model will be evaluated as a mixed-methods study; further details of the evaluation protocol are defined in a separate publication.[10]

We aim to bring the online learning component (MOOC) together with a face-to-face university level course, to enhance the learning experience and to challenge and enrich postgraduate level course taught with traditional methods. This will allow diversifying knowledge delivery mediums and will enable university students to be exposed to a global community of learners. The MOOC will allow learners the flexibility of amending the learning from any location, the advantage of meeting a large number of students outside their regular university classrooms and will meet EIT Health's call for "Massive dissemination" of innovative education ideas. A blended course will allow knowledge integration between the university students and the global learners joining the MOOC from all over the world, which can add richness and enhance the learning experience for all learners. The limited availability of courses on RWE, a topic that is gaining popularity in healthcare and the use of the latest advances in online education (blended MOOC) is what makes our proposed programme unique regarding the topic and educational strategies used.

METHODS AND ANALYSIS

MOOC Overview

The project will deliver an education programme via a MOOC and a face to face course, to create a blended format for postgraduate students and professionals interested in the application of RWE data analysis and in furthering their knowledge base and skill set to include conducting and commissioning RWD analysis. To help meet this goal, a key objective of the MOOC is to establish a global network of people to continue and advance the dialogue on data science in healthcare. It is expected that the MOOC will provide the opportunity to understand the fundamentals of RWE data science and to implement methods for analysing RWD.

MOOC development

The course will be mainly organized from Imperial College's Global eHealth Unit and course material will be filmed on Imperial College premises. The face-to-face component of the course will be offered at the University of Oxford. The course will be delivered in five weeks (5 modules). However, the fifth module will only be offered to learners who sign up for certification. The course

For peer review only - http://bmjopen.bmj.com/site/about/guidelines.xhtml

will be offered on specific dates and will be run for 5 weeks, and each module will be released on a weekly basis. Pilot implementation of the course will occur in 2018; partners will construct the core course programme, developing the face-to-face course and the complementary digital learning. Two face-to-face workshops will be held in the UK to pilot the blended course design. Scale-out will occur in 2019, face-to-face workshops will be held in France, Sweden, Spain and the UK, one per quarter. This will expand the reach of the programme and facilitate project development which will be done in conjunction with industry partners in each EU region. Workshops will include industry partners focused on the design, development and implementation of RWE methods

MOOC Pedagogical design

Course design will be based on the hybrid connectivist-heutagogical learning theories. The connectivism theory emphasizes the use of networking and communication to deliver the learning material, where interactions and discussions between learners can also contribute to the introduction of new knowledge [11]. Heutagogy principles emphasizes creating competent learners through touching on “learner autonomy and self-directedness.”[12] To enhance the connectivist characteristics of course, the digital platform will be created in a way that can enable learners to create social networks and communicate with like-minded individuals. One of the objectives of the course is to establish a global network to continue and advance the dialogue on data science in healthcare. Attendance in a course with healthcare professionals from around the world will give the postgraduate students the opportunity to appreciate the role of data in real life clinical settings and introduce them to methods and challenges faced in practical healthcare settings. At the same time, healthcare professionals joining the course will be able to communicate and engage with other professionals who will be possible using data in a different healthcare setting; clinical or non-clinical. The heutagogical characteristics of the course will be enhanced, by allowing learners the flexibility to go through the course material in their own time and to move through the material according to their preference. This learning principle will also be enhanced by incorporating social media into the discussions, [12] and creating a course hashtag that can be accessed by learners for further discussion.

Teaching strategies

The course plan will be prepared following the ADDIE model (instructional design model) consisting of Analysis, Design, Development, Implementation and Evaluation.[13] The ADDIE model provides a systematic, step-by-step framework to ensure the course is developed in a structured and a comprehensive way.[14] To teach innovation, learners will be structured into teams to develop and implement a data science project. These initiatives will be RWE projects that will address current health problems. Our assessment strategies will include both formative (polls and peer-assessment) and summative assessments (multiple choice questions/module). We will offer an optional fee-based certificate for completion of the MOOC and a separate project. The project will involve the proposal of an RWE data analysis methodology (using techniques learned in the course) to solving a current healthcare problem. Learners will be engaged in collaborative exercises and reflective discussions with other learners where the ‘teacher’ will act as a facilitator and a contributor to the discussions. Each module will provide a list of required reading, recommended reading, and RWE related datasets and tools to allow learners to individualise their learning and explore other sources of knowledge.[15]

For peer review only - http://bmjopen.bmj.com/site/about/guidelines.xhtml

Target Learners

The course will cater to two types of learners: current university level students (postgraduate) and professionals in the healthcare field (e.g. professionals working in healthcare, commissioners, policy makers, healthcare regulators and those working in the life sciences.); (1) who have some background knowledge of statistics in healthcare and/or (2) who carry out hands-on data analysis in their work or studies. The target audience will include postgraduate students in health-related fields with an interest in the use of RWD and RWE data science in healthcare and professionals working in NHS commissioning, healthcare regulation and life sciences. Selection parameters will be based on participant's occupation and statistical knowledge. Participants who are postgraduate students or professionals in healthcare fields, who have completed a basic university-level statistics course or who have professional experience in data analysis and research will be eligible to join the MOOC. The required prerequisite is knowledge in basic statistics and understanding of statistical measures used in healthcare, which is met if the learner completed a university-level basic statistics course or professionally works on data analysis.

Learning outcomes

The content from the course develops skills in the context of RWE, including frameworks for analysing and evaluating content (data) and subsequently, will encourage them to embark on healthcare RWE related projects, innovation and entrepreneurship. To complete the course, learners will be structured into teams to develop and implement a data science project. These initiatives will be RWD projects that will address current healthcare problems. Completion of projects will be a form of learning into practice and establish a foundation for further commercial activity or research. The proposed blended format will prepare future (postgraduate students) and current healthcare professionals with the required skills and knowledge to conduct and be part of RWE projects and will help develop them to the growing need of data analysis skills in the healthcare field. Topics and key concepts will include Principles of RWE in Health, Information Governance for RWE, Design, Methodology and Framework for RWE, and RWE Exploratory Analysis and Evaluation.

One of the primary learning outcomes of the course will be the ability to apply an established RWE framework that ensures transparency and integrity around the collection, analysis and use of RWD. Other learning outcomes include: (1) To provide learners with formal knowledge of RWE, (2) To provide learners with experiences that expand upon their existing knowledge of RWE, (3) To have learners determine how to learn about the evolving topic, (4) To have learners self-organise into social networks to develop knowledge and address situated problems, (5) To have learners collaborate to master the competencies required to solve RWD challenges. Each learning outcome will be delivered on a module basis.

Production

A pan-European collaboration has been established including Université Grenoble Alpes, Karolinska Institutet, Imperial College London and the University of Oxford. Video presentations will be made with partners and recorded and edited by the production team. Written pieces such as articles, case studies powerpoint presentations, quizzes and assessment will be developed by the production

For peer review only - http://bmjopen.bmj.com/site/about/guidelines.xhtml

team and partners prior to integration onto the delivery platform. The face-to-face, the on-campus course will be developed and offered at the Medical Sciences Division at the University of Oxford. To evaluate the content before it is released we will run a beta trial of the MOOC with a sample of target learners.

Learner Recruitment

We will develop a marketing campaign focused on social media and online advertising in order to recruit learners; also coordinating with the marketing of other courses. We will also cross market to our database of 1000+ learners. Specifically, course promotion will involve a variety of methods to include (1) the Institution's own website (2) social media (e.g. Facebook, Twitter, Google+, LinkedIn) (3) posting of articles via Distance Learning Portals (4) materials such as flyers, brochures, roll-ups and posters (5) email mailings (6) regular course newsletters. Providing the recruiting process on public platforms will ensure the process is clear, open and transparent. As a key element of the course will be connectivity between participants, learners will be encouraged to share knowledge, experience and ideas throughout the course by discussion forums and via social media. We will continue the same promotion activities during the course delivery and to actively encourage partition, newsletter, and course social media hashtag will be available during and one year is the course.

Problems anticipated

To create a feasible work plan, potential risks and contingencies were identified in the following areas:

Instructional design:

Key risks:

on-boarding of new staff

Contingency:

Imperial College is contributing existing staff familiar with this form of course design.

Learning management system design:

Key risks:

Requesting functionality not possible from the LMS

Contingency:

Will mitigate risk through early engagement with faculty and planning in advance.

The beta trial of course:

Key risks:

Non-availability of test students

Contingency:

Will incentive beta participant learners

Live implementation of course:

Key risks:

Student recruitment and marketing

Contingency:

Will market the course from the beginning of the year to ensure high student enrolment numbers

For peer review only - http://bmjopen.bmj.com/site/about/guidelines.xhtml

Evaluation:

Key risks:

Ethical considerations in the evaluation

Contingency:

Will seek ethical approval of the evaluation process

MOOC Outcomes

Success for this course will be maintaining an active cohort of learners who will engage in continued discussion on these topics (e.g. via social media), where we also establish a basis for possible collaborations on future initiatives. The short-term impact of the course includes enabling participants to join networks of data science in healthcare and engaging and educating participants on the basic skills needed to conduct RWE projects in their work or studies. The long-term impact includes enabling participants to maintain their presence in networks of data science in healthcare and enabling participants to conduct RWE projects in their work and contribute to the improvement of healthcare through the use of data and evidence-based approaches.

We expect learners to take skills taught in the MOOC and use them to seek new employment or start to initiatives in these domains. Learners will be able to start new RWE initiatives as a result of the practical RWE projects they will complete in the MOOC. Learners will submit a project using an RWE framework taught in the MOOC that will be peer-assessed. To make sure that projects are assessed properly, a rubric with clearly defined guidelines and reviewing criteria will be provided. The course coordinator will also facilitate these exercises. The successful completion of the RWE project (both submitting a project and peer assessing the project) will give learners the basic skills to carry out an RWE project.

Evaluation

MOOC evaluation is a significant practice to assess its value and effectiveness. [16] To evaluate the course's impact, we will complete an evaluation on a participant-level to measure the continuation of MOOC effects over me. We plan to prepare the evaluation findings in the form of a report that can be shared with our partners. The evaluation will be mainly used to evaluate the impact of the current project learners became involved in and its success in delivering the intended outcomes. However, the evaluation will also provide insights that can be learned to enhance future iterations of the MOOC. To ensure evaluation results are shared and used for the improvement of future projects, a report summarising the research findings of our MOOC evaluation will be published in a peer-reviewed journal. Findings shall also be disseminated at an international conference.

During the course launch, we will use xAPI to track all learning activity taking place in the course. This software tracks and records all learning activity with the learning management system and will be used to assist to provide real-me feedback to the course organisers on how learners are responding to the course. Moreover, pre-MOOC and post-MOOC survey data will be captured to evaluate learner's satisfaction with the course, and whether or not it met their expectations. Course metrics such as retention on the course, number of participants in discussion posts, number of participants in the course's social media posts and the number of learners who completed the RWE projects will all be used as indicators for course impact.

For peer review only - http://bmjopen.bmj.com/site/about/guidelines.xhtml

Other post-project monitoring will be conducted with learners from the MOOC via 2 interviews, the first 1 month are the course, the second, 3 months post-course. We are aiming to recruit 16 participants for interviews and if more than 16 agree to an interview, we will include participants who represent as wide a range of data science backgrounds and levels of MOOC completion as possible (thereby liming demographic duplication and the possible number of individuals to interview). We have targeted 16 learners because based on the literature, [17] a study size of 16 is appropriate for collection of qualitative data and for providing sufficient insight into the questions studied. However, should there be a need for further exploration of phenomena under study (and themes and findings which emerge), we will attempt to continue interviewing participants until saturation is reached on the most important themes relating primarily to the course's effectiveness

A mixed methods approach will be used to analyse these outcomes, incorporating pre-course and post-course survey results, semi-structured interviews of a subset of the learners and social media network analysis. The evaluations main focus will be learners' application of the practical skills gained through the MOOC in their workplace using Kirkpatrick evaluation method and will be conducted on selected learners a year post-implementation.[18] The Kirkpatrick evaluation is a methodology that evaluates the success of training courses, and it is a suitable choice for evaluating the success of the proposed MOOC since the MOOC's aim is to teach hands-on skills in RWE data analysis.[18] In addition, course metrics such as retention on the course, number of learners who completed the RWE projects will all be used as indicators of course outcomes achievement.

Patient and Public Involvement

Members of the public informed the development of research questions and study objectives via a workshop held at the European Scientific Institute in July 2017. Learners participating in this study completed informed consent and shall receive a copy of results and publications from this work.

Sustainability

We plan to sustain the course and course updates via internal funding following this investment round and sustaining the course through course certification fees.

ETHICS AND DISSEMINATION

The protocol for the course was approved by EIT Health (Grant 18654) as part of the EIT Health CAMPUS Deferred Call for Innovative Education 2018. Results will be published in a peer-reviewed journal.

Acknowledgements

This work was funded by EIT Health (Grant 18654). This work was also supported by the Sir David Cooksey Fellowship in Healthcare Translation, the Oxford Academic Health Sciences Centre and the SENS Research Foundation.

For peer review only - http://bmjopen.bmj.com/site/about/guidelines.xhtml

Contributorship Statement

EM conceived the study and drafted the manuscript. AA revised the manuscript, structured it to journal requirements and made amendments based on feedback from EM. JC contributed to the protocol design and provided feedback on the manuscript iterations. AC, GW revised iterations of the pre-publication protocol and provided feedback for amendment. DB revised all versions, structured the project workplan with EM and provided methods feedback. All authors read/edited the first and second drafts and approved the final manuscript. EM responded to all external peer review comments. EM is the guarantor. All authors read and approved the final manuscript. All authors completed the ICMJE uniform disclosure form at www.icmje.org/coi_disclosure.pdf. There are no relevant conflicts of interest, financial or other types of relationships that may influence the manuscript declared by authors. Authors do not have any patents and are not associated to any conditions or circumstances that may lead to conflicts of interest.

Funding statement

This work was funded by EIT Health (Grant 18654).

For peer review only

REFERENCES

1. Wang Y, Kung L, Byrd TA. Big data analytics: Understanding its capabilities and potential benefits for healthcare organizations. *Technological Forecasting and Social Change* 2018;**126**:3–13. doi:10.1016/j.techfore.2015.12.019
2. American Management Association (AMA). Conquering Big Data: Building Analytical Skills in Your Organization. American Management Association Press 2013. https://cdns3.trainingindustry.com/media/16687264/conquering_big_data_survey_final.pdf (accessed 2 Jul 2018).
3. Sherman RE, Anderson SA, Dal Pan GJ, *et al.* Real-world evidence—what is it and what can it tell us. *N Engl J Med* 2016;**375**:2293–2297.
4. Hubbard TE, Paradis R, The Network for Excellence in Health Care Innovation. The Network for Excellence in Health Innovation: Issue Breif: Real World Evidence: A New Era for Health Care Innovation. One Broadway, Cambridge, MA 02142: : The Network for Excellence in Health Innovation 2015. https://www.nehi.net/writable/publication_files/file/rwe_issue_brief_final.pdf (accessed 2 Jul 2018).
5. Steels L. The Coming of MOOCs. In: *Volume 6: Music Learning with Massive Open Online Courses (MOOCs)*. IOS Press 3–20.
6. Hossain MS, Shofiqul Islam M, Glinsky JV, *et al.* A massive open online course (MOOC) can be used to teach physiotherapy students about spinal cord injuries: a randomised trial. *J Physiother* 2015;**61**:21–7. doi:10.1016/j.jphys.2014.09.008
7. George PP, Papachristou N, Belisario JM, *et al.* Online eLearning for undergraduates in health professions: A systematic review of the impact on knowledge, skills, attitudes and satisfaction. *J Glob Health* 2014;**4**:010406. doi:10.7189/jogh.04.010406
8. Barnes C. MOOCs: The Challenges for Academic Librarians. *Australian Academic & Research Libraries* 2013;**44**:163–75. doi:10.1080/00048623.2013.821048
9. Israel MJ. Effectiveness of Integrating MOOCs in Traditional Classrooms for Undergraduate Students. *The International Review of Research in Open and Distributed Learning* 2015;**16**. doi:10.19173/irrodl.v16i5.2222
10. Meinert E, Alturkistani A, Brindley D, *et al.* Protocol for a Mixed-Methods Evaluation of a Massive Open Online Course on Real World Evidence. *BMJ Open.*
11. Duke B, Harper G, Johnston M. Connectivism as a digital age learning theory. *The International HETL Review* 2013;**2013**:4–13.
12. Blaschke LM. Heutagogy and lifelong learning: A review of heutagogical practice and self-determined learning. *The International Review of Research in Open and Distributed Learning* 2012;**13**:56–71.

For peer review only - http://bmjopen.bmj.com/site/about/guidelines.xhtml

13. Kruse K. An Introduction to the ADDIE Model for Instructional Designers. E-Learning Heroes. 2002.https://community.articulate.com/articles/an-introduction-to-the-addie-model-for-instructional-designers (accessed 2 Jul 2018).
14. Peterson C. Bringing ADDIE to Life: Instructional Design at Its Best. *Journal of Educational Multimedia and Hypermedia* 2003;**12**:227–41.
15. Manallack DT, Yuriev E. Ten Simple Rules for Developing a MOOC. *PLOS Computational Biology* 2016;**12**:e1005061. doi:10.1371/journal.pcbi.1005061
16. Chapman SA, Goodman S, Jawitz J, *et al.* A strategy for monitoring and evaluating massive open online courses. *Evaluation and Program Planning* 2016;**57**:55–63. doi:10.1016/j.evalprogplan.2016.04.006
17. Adler P, Adler P. Expert voices. In Baker SE, Edwards R, Doidge M. How many qualitative interviews is enough?: Expert voices and early career reflections on sampling and cases in qualitative research. 2012.
18. Kirkpatrick DL. *Evaluating Training Programs: The Four Levels*. ReadHowYouWant.com 2009.

For peer review only

For peer review only - http://bmjopen.bmj.com/site/about/guidelines.xhtml
