# Supplementary material for: Real-world evidence for postgraduate students and professionals in healthcare: protocol for the design of a blended massive open online course
Source: BMJ Open. 2018 Oct 4;8(9):e025196. doi: 10.1136/bmjopen-2018-025196 (PMC6173253; doi:10.1136/bmjopen-2018-025196)
Supplement: Reviewer comments [file bmjopen-2018-025196.reviewer_comments.pdf]

PEER REVIEW HISTORY

BMJ Open publishes all reviews undertaken for accepted manuscripts. Reviewers are asked to complete a checklist review form (**http://bmjopen.bmj.com/site/about/resources/checklist.pdf**) and are provided with free text boxes to elaborate on their assessment. These free text comments are reproduced below.

ARTICLE DETAILS

**TITLE (PROVISIONAL)**	Real World Evidence for postgraduate students and professionals in healthcare: protocol for the design of a blended Massive Open Online Course
**AUTHORS**	Meinert, Edward; Alturkistani, Abrar; Car, Josip; Carter, Alison; Wells, Glenn; Brindley, David

This article was not externally reviewed at BMJ Open. Protocols that have been independently assessed prior to submission to BMJ Open are usually fast-tracked to publication on the grounds that further substantial changes will not be possible. This independent assessment will usually be external, independent review for both a substantial grant award from a non-commercial or government funder and ethics approval.
